# Live Attenuated *Francisella novicida* Vaccine Protects against *Francisella tularensis* Pulmonary Challenge in Rats and Non-human Primates

**DOI:** 10.1371/journal.ppat.1004439

**Published:** 2014-10-23

**Authors:** Ping Chu, Aimee L. Cunningham, Jieh-Juen Yu, Jesse Q. Nguyen, Jeffrey R. Barker, C. Rick Lyons, Julie Wilder, Michelle Valderas, Robert L. Sherwood, Bernard P. Arulanandam, Karl E. Klose

**Affiliations:** 1 South Texas Center for Emerging Infectious Diseases and Department of Biology, University of Texas San Antonio, San Antonio, Texas, United States of America; 2 Infectious Disease Research Center, Colorado State University, Fort Collins, Colorado, United States of America; 3 Applied Life Sciences & Toxicology, Lovelace Respiratory Research Institute, Albuquerque, New Mexico, United States of America; Emory University School of Medicine, United States of America

## Abstract

*Francisella tularensis* causes the disease tularemia. Human pulmonary exposure to the most virulent form, *F. tularensis* subsp. *tularensis* (Ftt), leads to high morbidity and mortality, resulting in this bacterium being classified as a potential biothreat agent. However, a closely-related species, *F. novicida*, is avirulent in healthy humans. No tularemia vaccine is currently approved for human use. We demonstrate that a single dose vaccine of a live attenuated *F. novicida* strain (Fn *iglD*) protects against subsequent pulmonary challenge with Ftt using two different animal models, Fischer 344 rats and cynomolgus macaques (NHP). The Fn *iglD* vaccine showed protective efficacy in rats, as did a Ftt *iglD* vaccine, suggesting no disadvantage to utilizing the low human virulent *Francisella* species to induce protective immunity. Comparison of specific antibody profiles in vaccinated rat and NHP sera by proteome array identified a core set of immunodominant antigens in vaccinated animals. This is the first report of a defined live attenuated vaccine that demonstrates efficacy against pulmonary tularemia in a NHP, and indicates that the low human virulence *F. novicida* functions as an effective tularemia vaccine platform.

## Author Summary

*Francisella tularensis* is a bacterium that causes the infectious disease tularemia. *F. tularensis* has been developed as a biothreat agent, because it causes high morbidity and mortality when spread by aerosol. There is currently no approved vaccine for human use, making mankind vulnerable to the illicit use of this organism. *F. tularensis* contains a cluster of genes in the Francisella Pathogenicity Island (FPI) that are required for replication inside host macrophages and virulence. In the current study we created a live vaccine strain by inactivating an FPI gene, *iglD*, in a closely-related species that does not cause disease in humans, *F. novicida* (Fn *iglD*). We demonstrate that vaccination with Fn *iglD* protects against exposure to airborne *F. tularensis*. Fn *iglD* vaccination induces antibody and cellular immune responses and protects two different animals, rats and non-human primates, against lethal pulmonary tularemia challenges. These two animal models reflect human sensitivity to *F. tularensis*. Our results suggest that a vaccine made from the low virulence *F. novicida* will protect humans against aerosol exposure to this dangerous pathogen.

## Introduction

*F. tularensis* is a highly infectious bacterium that causes tularemia in humans, a disease that has a high mortality rate when acquired through the pulmonary route. *F. tularensis* is able to survive and replicate within host macrophages, and this ability is essential for its virulence. Within macrophages, *F. tularensis* escapes from the phagosomal compartment and replicates within the cytosol [1]. Phagosomal escape is mediated by a cluster of virulence genes in the Francisella Pathogenicity Island (FPI) that encode a Type VI-like secretion system [2]. *F. tularensis* acquired through the pulmonary route disseminate to tissues outside the lung, where they replicate to high levels within internal organs such as the liver. Early in infection, *F. tularensis* appears to induce broad immunosuppression within the host [3], as proinflammatory cytokine expression is notably repressed [4] and infected cells are unable to respond to TLR-dependent secondary stimuli [5]. *F. tularensis* subsp. *tularensis* (Ftt) exhibits the highest level of virulence in all mammalian hosts, including humans, and because of the morbidity and mortality associated with disease as well as the potential for aerosol dissemination, it has been designated a category A biothreat agent. A closely-related species, *F. novicida* (Fn), is considered essentially avirulent for healthy humans and for this reason is exempt from select agent status.

There is currently no tularemia vaccine approved for human use. A live attenuated vaccine strain (LVS) was derived in Russia by repeated passage of *F. tularensis* subsp. *holarctica* (Fth). LVS vaccination can protect against pulmonary challenge with Ftt in rats [6], rhesus macaques and humans [7]. The LVS genome contains a large number of mutations that distinguish it from other Fth strains, but the primary attenuating mutations appear to be the deletion of a lipoprotein (FTT0918) and a pilus subunit (*pilA*) [8]. Questions of stability, reversion frequency, and levels of protection may prevent LVS from becoming licensed for human use. However extensive studies with LVS have illuminated attributes of protective immunity against tularemia in mice. T-cell mediated immunity has been shown to be critical, but antibodies also appear to play a role; despite this, no specific correlate of protection has been established (for review of this extensive field, please see [9]). The efficacy of LVS suggests that a safe rationally designed live attenuated vaccine would be effective against pulmonary tularemia.

Mice have traditionally been the preferred model for tularemia vaccine development, due to ease of use, availability of reagents, and sensitivity to *F. tularensis* infections. However, tularemia vaccine development has been hampered by the extreme sensitivity of mice to *Francisella* subspecies, such that it has proven difficult to induce even partial protection against pulmonary Ftt exposure [10]. In fact, both LVS and Fn, which are known to be essentially avirulent in healthy humans, are virulent in mice. Recently, the Fischer 344 rat has been promoted as a better tularemia vaccine model, in that the rat shows similar sensitivities to the various *F. tularensis* strains as humans [6], [11]. Non-human primate models of tularemia have also been reported, including rhesus macaques, marmosets, and African green monkeys [12]–[14]. Cynomolgus macaques are also sensitive to pulmonary infection by Ftt, which causes a fatal systemic disease similar to that seen in humans [15].

In the current study, we demonstrate the protective efficacy of a live highly attenuated Fn strain (Fn *iglD*) against pulmonary infection with Ftt, in both rats and cynomolgus macaques. Our results suggest that the live attenuated Fn *iglD* strain is a vaccine platform that is inherently safer, yet still effective for protecting humans against pulmonary tularemia.

## Results

### Fn *iglD* vaccination protects rats against Ftt pulmonary challenge

The FPI is required for intramacrophage growth and virulence of *Francisella* spp. [16], and inactivation of most FPI genes renders *Francisella* spp. highly attenuated for intracellular growth and for virulence in animals. The FPI protein IglD is required for *Francisella* phagosomal escape and intramacrophage replication within macrophages [17]–[19] and *Francisella iglD* mutant strains are highly attenuated for virulence in mice [20], [21]. We constructed Fn and Ftt *iglD* mutant strains and confirmed that both were defective for intramacrophage replication and virulence in mice, in contrast to the respective wildtype strains (Fig. S1; Ftt has 2 identical copies of *iglD*, and is thus a *iglD1 iglD2* mutant).

In preliminary studies, the Fn *iglD* strain inoculated intranasally into mice was able to fully protect against subsequent pulmonary challenge with a relatively high dose of the wildtype *F. novicida* strain (10^3^ CFU), but was unable to provide any protection against pulmonary challenge with a similar dose of Ftt (10^3^ CFU; Table 1). However, the Ftt *iglD* strain inoculated intranasally into mice was also unable to provide protection against pulmonary challenge with a similar dose of Ftt (10^3^ CFU). This suggests that the failure of Fn *iglD* vaccination to protect against Ftt pulmonary challenge in mice is not due to some inherent deficiency in Fn, but rather may be due to the mouse being an inappropriate animal model for tularemia vaccine studies because of the extreme sensitivity of mice to all *Francisella* subspecies.

**Table 1:** Vaccination of mice with Fn *iglD* or Ftt *iglD* does not protect against Ftt challenge.

Intranasal Vaccination (strain)	Vaccine dose (CFU)	Survival (30 days)	Intranasal Challenge (strain, CFU)	Survival (30 days)
Fn *iglD*	9.7×10^8^	5/5	Fn 10^3^	5/5
Mock (PBS)	-	-	Fn 10^3^	0/5
Fn *iglD*	9.7×10^8^	5/5	Ftt 10^3^	0/5
Ftt *iglD*	4.8×10^6^	5/5	Ftt 10^3^	0/5

**Figure 1:**
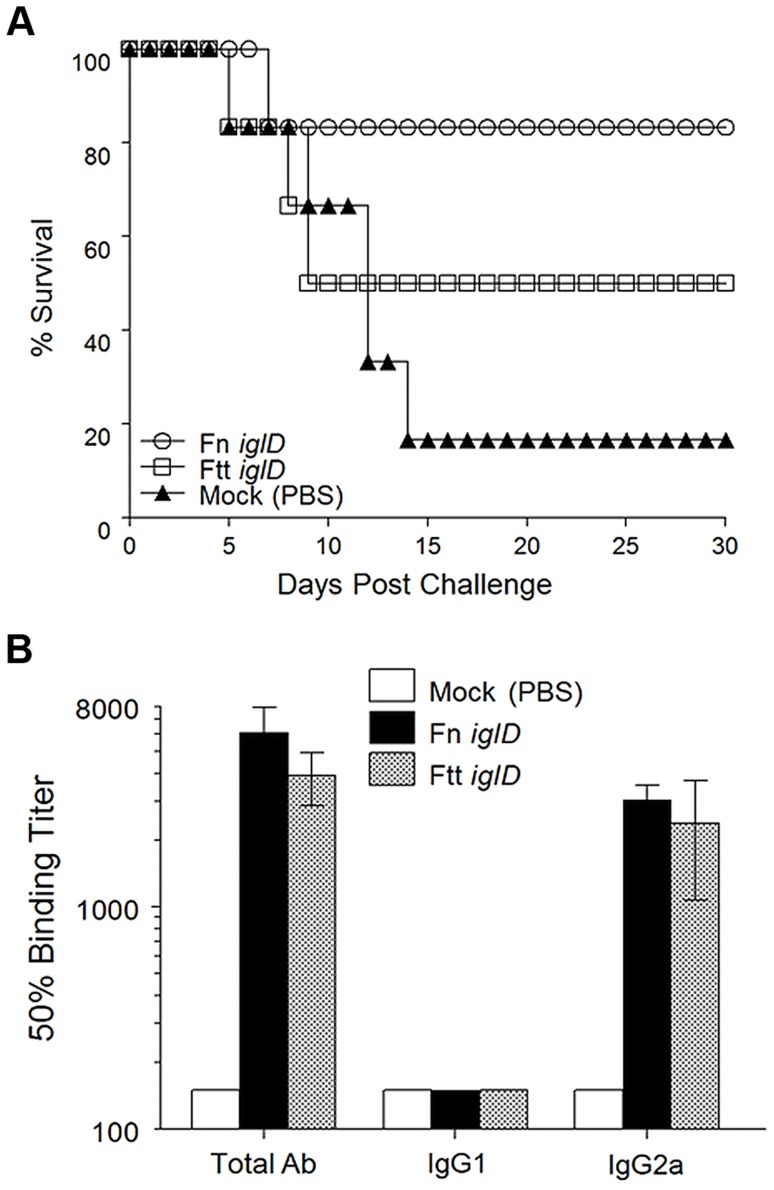
Fn *iglD* vaccination is protective against pulmonary Ftt challenge in Fischer 344 rats. **A.** Groups of Fischer 344 rats (6 rats/group) were inoculated orally with 10^7^ CFU Fn *iglD* (open circles) or Ftt *iglD* (open squares), or mock vaccinated (filled triangles). Rats were challenged 30 days post vaccination with 10^4^ CFU Ftt delivered intratracheally, and monitored for survival. Difference in survival was significant for Fn *iglD*-vaccinated rats compared to mock vaccinated (*P* = 0.0439; Kaplan-Meier). Difference in survival of Ftt *iglD*-vaccinated rats compared to mock vaccinated was not significant. **B.** Sera from vaccinated rats were analyzed 30 days post-vaccination for Fn- or Ftt-specific antibodies (total Ab, IgG1, and IgG2a).

The Fischer 344 rat has been promoted as a relevant animal model for tularemia vaccine studies, due to the relative sensitivities of the rat to the various *Francisella* subspecies, which mirror human sensitivities [6], [11]. Moreover, LVS vaccination of Fischer 344 rats protects against pulmonary exposure to Ftt [6], [22]. We have previously shown that oral vaccination of Fischer 344 rats with attenuated Fn strains induces comparable levels of protection against pulmonary Ftt challenge as intratracheal vaccination [23], so the oral route was used in the following experiments. To determine the relative efficacies of Fn and Ftt live vaccine platforms in the rat, we vaccinated Fischer 344 rats (n = 6) orally with either Fn *iglD* or Ftt *iglD* (both at 10^7^ CFU) and then challenged the vaccinated rats 30 days later with Ftt (10^4^ CFU) delivered intratracheally (Fig. 1A). 5 of 6 Fn *iglD* vaccinated rats (83%) survived pulmonary Ftt challenge. 3 of 6 Ftt *iglD*-vaccinated rats (50%) survived pulmonary Ftt challenge. These results demonstrate that there is no disadvantage to utilizing Fn instead of Ftt as the platform for live attenuated vaccines against pulmonary Ftt. Only one mock-vaccinated rat (n = 6) survived pulmonary challenge with Ftt.

Measurement of serum antibody levels in vaccinated rats revealed similar levels of Fn- or Ftt-specific antibodies in both groups, constituted by high levels of IgG2a and low levels of IgG1 (Fig. 1B); this polarized Th1-type response has been reported previously in immunized rats [11].

### Pulmonary Fn *iglD* vaccination induces protective immunity against Ftt pulmonary challenge in rats

**Figure 2:**
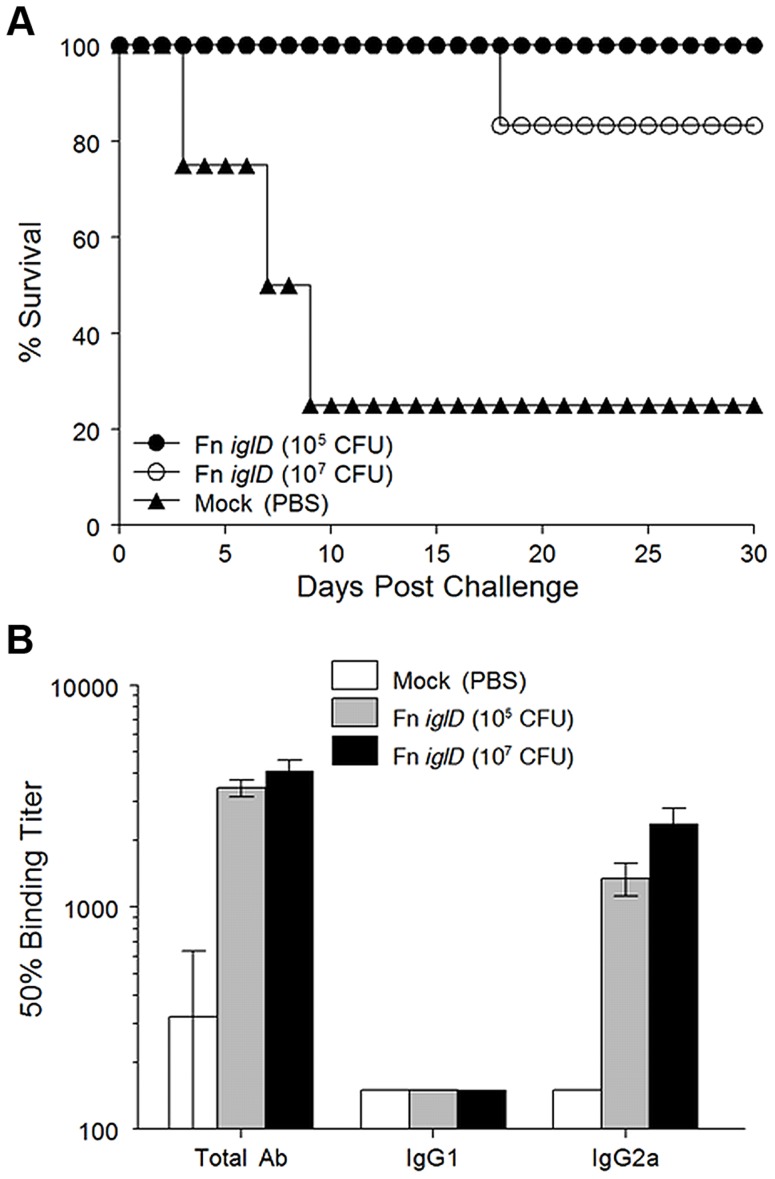
Fn *iglD* pulmonary vaccination protects Fischer 344 rats against pulmonary Ftt challenge. **A.** Groups of Fischer 344 rats were inoculated intratracheally with 10^5^ (filled circles; n = 4) or 10^7^ CFU (open circles; n = 6) Fn *iglD* or mock-vaccinated (filled triangles; n = 4). 30 days post vaccination rats were challenged with 10^4^ CFU Ftt delivered intratracheally, and monitored for survival. Difference in survival of Fn *iglD*-vaccinated rats compared to mock vaccinated was significant (*P* = 0.0455 for 10^5^ CFU and p = 0.0330 for 10^7^ CFU; Kaplan-Meier). Due to shortage of rats, two group sizes were smaller than optimal n = 6 [6]. **B.** Sera from vaccinated rats were analyzed 30 days post-vaccination for Fn-specific antibodies (total Ab, IgG1, and IgG2a).

Since oral vaccination of rats with the Fn *iglD* live vaccine strain was shown to induce protective immunity against pulmonary Ftt challenge, we determined whether pulmonary vaccination of rats with Fn *iglD* also induced protective immunity against pulmonary Ftt challenge. Fischer 344 rats were vaccinated intratracheally with Fn *iglD* at 10^5^ (n = 4) or 10^7^ (n = 6) CFU, and challenged 30 days later with Ftt (10^4^ CFU) delivered intratracheally (Fig. 2A). All rats vaccinated with Fn *iglD* at 10^5^ CFU (100% protection) and 5/6 (83%) of rats vaccinated with Fn *iglD* at 10^7^ CFU survived pulmonary challenge with Ftt, whereas only one of four mock-vaccinated rats survived this challenge, demonstrating the efficacy of pulmonary vaccination with Fn *iglD* to protect against pulmonary challenge with Ftt.

Measurement of serum antibody levels in Fn *iglD*-vaccinated rats (via pulmonary route) again revealed a polarized response similar to oral vaccination, with high levels of Fn-specific IgG2a and low levels of IgG1 (Fig. 2B). [11] It is known that a major target of the humoral response to *Francisella* infection is the O antigen (OAg) of the LPS, and that Fn and Ftt express distinct OAgs [24]. LVS expresses an OAg that is indistinguishable from the OAg of Ftt [25]. To determine if a humoral response to OAg was induced in vaccinated rats, we performed Western immunoblot analyses of serum from one of the rats immunized intratracheally with Fn *iglD* (10^7^ CFU) and compared that to the reactivity of serum from a rat immunized in a previous study by the same route with the same inoculum of LVS [11], (Fig. S2). Serum from the rat vaccinated with Fn *iglD* reacted strongly with purified LPS from Fn, but also with LPS from LVS. In contrast, the serum from an LVS-vaccinated rat reacted strongly with LVS LPS and did not react at all with Fn LPS. Interestingly, the reactivity of both rat sera was predominantly to high molecular weight material, likely the OAg capsule [26]. These results confirm that the OAg of Fn and LVS/Ftt are distinct and demonstrate that in rats, just as in mice [24], humoral responses directed towards LVS/Ftt OAg do not crossreact with Fn OAg. However there was some recognition of the LVS/Ftt OAg in Fn *iglD*-vaccinated rats.

In order to measure the cellular responses of rats vaccinated with Fn *iglD*, we vaccinated 3 rats by the intratracheal route at 10^7^ CFU, collected spleens at 28 days post vaccination, and measured IFNγ production upon stimulation with increasing dose of UV-inactivated Fn *iglD*,(10^5^ and 10^6^ CFU) or the (irrelevant) antigen HEL (Fig. S3). The Fn *iglD*-vaccinated rats exhibited a dose-dependent increase (p<0.05) in cellular responses to Fn *iglD*, whereas mock vaccinated rats showed no cellular responses to Fn *iglD*.

### Pulmonary Fn *iglD* vaccination induces protective immunity against Ftt pulmonary challenge in cynomolgus macaques

The cynomolgus macaque is sensitive to pulmonary infection with Ftt [15], with an LD_50_ of approximately 1 CFU via the aerosol route. Clinical symptoms of infection in this model include high respiration rates and serum C-reactive protein (CRP) levels, with corresponding high bacterial burdens in the lungs and tracheobronchial lymph nodes (see below). The cynomolgus macaque has been proposed as a relevant non-human primate (NHP) model for tularemia vaccine development. We determined whether pulmonary vaccination of cynomolgus macaques with Fn *iglD* induced protective immunity against pulmonary Ftt challenge. 6 cynomolgus macaques were vaccinated via bronchoscopy with Fn *iglD* at 10^8^ CFU, and an additional 4 control animals were mock vaccinated with PBS. 4 additional NHPs received LVS vaccination. Because LVS vaccination is known to induce protective immunity against Ftt in humans when administered through the skin, these animals were vaccinated by the subcutaneous route to serve as a vaccination standard against which the Fn *iglD* vaccine could be compared. The Fn *iglD* strain was well-tolerated in vaccinated NHPs, similar to the LVS vaccine, based on the lack of increase in respiration rate, and low serum CRP levels.

**Figure 3:**
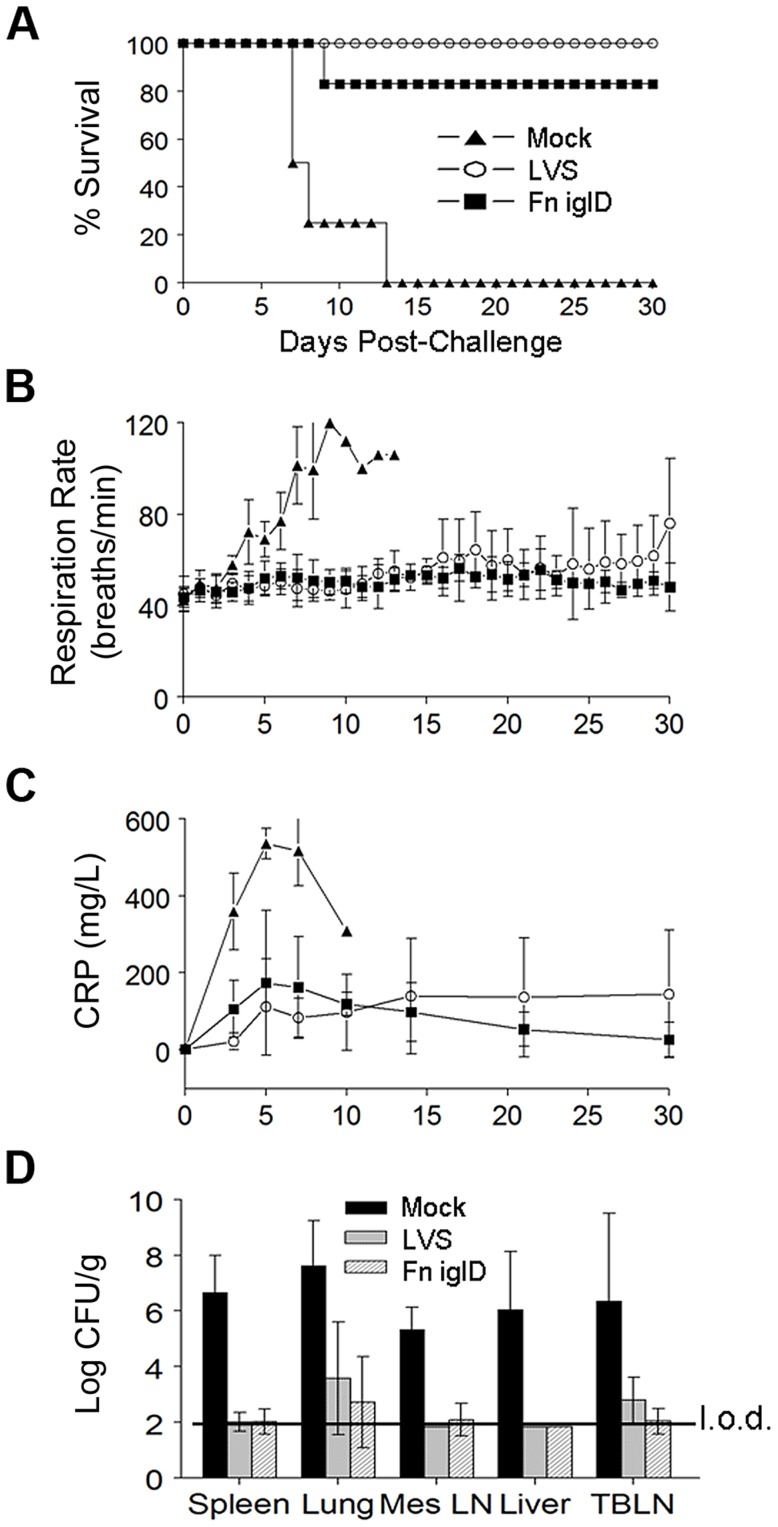
Fn *iglD* vaccination protects NHP against subsequent pulmonary challenge with Ftt. **A.** Groups of cynomolgus macaques were vaccinated with 10^8^ CFU Fn *iglD* delivered via bronchoscopy (n = 6; open circles), or 10^8^ CFU LVS delivered subcutaneously (n = 4; filled squares), or mock vaccinated (n = 4; filled triangles). 35 days post vaccination NHP were challenged with ∼1000 CFU Ftt via head-only aerosol inhalation; actual presented doses were determined (Table S1). NHP were monitored at intervals shown for 30 days post-challenge for (**A**) survival, (**B**) respiration rates, and (**C**) serum CRP levels. Difference in survival was significant for Fn *iglD*- and LVS-vaccinated NHPs compared to mock vaccinated (*P* = 0.0048 and P = 0.0062 respectively; Kaplan-Meier). **D.** Bacterial burdens (Ftt) were determined in spleen, lung, mesenteric lymph nodes (MesLN), liver, and trachea-bronchial lymph nodes (TBLN) at the time of euthanasia (days 6–10 for mock vaccinees and the single Fn *iglD* vaccinated NHP that succumbed to pulmonary Ftt challenge [AO8070], day 30 for LVS- and surviving Fn *iglD*-vaccinated NHPs). The limit of detection (“l.o.d.”; depicted by line) was 70 CFU/g. No bacteria were recovered in the spleens or livers of any surviving Fn *iglD* vaccinated NHP; organ burdens of the individual vaccinated NHPs are shown in Fig. S5.

Vaccinated and control NHP were challenged 30 days later with Ftt delivered in a head-only aerosol chamber with presented doses of 2500–5000 CFU (Fig. 3A; presented doses for each NHP given in Table S1). Challenged NHP were monitored for a number of different parameters, including respiration rate, serum CRP levels, and disease symptoms. Mock vaccinated animals eventually exhibited severe disease symptoms that necessitated euthanasia of all 4 animals when moribund, at days 7 (2 X), 8, and 13 post Ftt challenge. In contrast, only one Fn *iglD* vaccinated animal required euthanasia when it became moribund at day 9 post challenge, and all other Fn *iglD* vaccinated NHPs survived to the end of the study at 30 days post challenge (83% protection). This demonstrates the efficacy of pulmonary vaccination with Fn *iglD* to protect against pulmonary challenge with Ftt in a NHP model of tularemia. All 4 LVS vaccinated NHPs also survived to the end of the study at 30 days post challenge (100% protection).

Mock vaccinated NHPs exhibited significantly increased respiration rates and serum CRP levels compared to the Fn *iglD*- and LVS-vaccinated NHPs beginning 3 days post-challenge with Ftt (Fig. 3B and 3C). Mock vaccinated NHPs also exhibited a trend in increased serum alanine transaminase (ALT), blood urea nitrogen (BUN), lactate dehydrogenase (LDH), and aspartate aminotransferase (AST) levels, indicating liver, kidney, and other tissue damage, although these levels did not reach statistical significance over those of the vaccinated NHPs, perhaps due to small sample size (Fig. S4). 2 of 4 mock-vaccinated animals had detectable bacteremia at days 5 and 6 post challenge, whereas none of the vaccinated animals had detectable bacteremia at any time post Ftt challenge. Bacterial organ burdens determined at autopsy revealed higher bacterial burdens in the spleen, lung, mesenteric lymph nodes, liver, and tracheo-bronchial lymph nodes of mock-vaccinated NHPs than in those of Fn *iglD*- and LVS-vaccinated NHP (Fig. 3D). In fact, 2/4 LVS-vaccinated and 3/6 Fn *iglD* vaccinated NHPs had no detectable bacterial burdens in any of the tissues sampled (limit of detection ∼70 CFU/g; Fig. S5).

When organ burdens of the individual Fn *iglD*-vaccinated NHPs were compared at autopsy, the single animal (AO8070) that succumbed to Ftt challenge (day 9) exhibited a high bacterial burden in the lung compared to the 5 NHPs that survived Ftt challenge (day 30) (Fig. S5). At the termination of the study, 2 LVS-vaccinated NHPs exhibited elevated bacterial burdens in the lung, elevated serum CRP levels, and elevated respiration rates (day 30) (Fig. S5), suggesting they may have progressed to terminal disease in an extended study.

**Figure 4:**
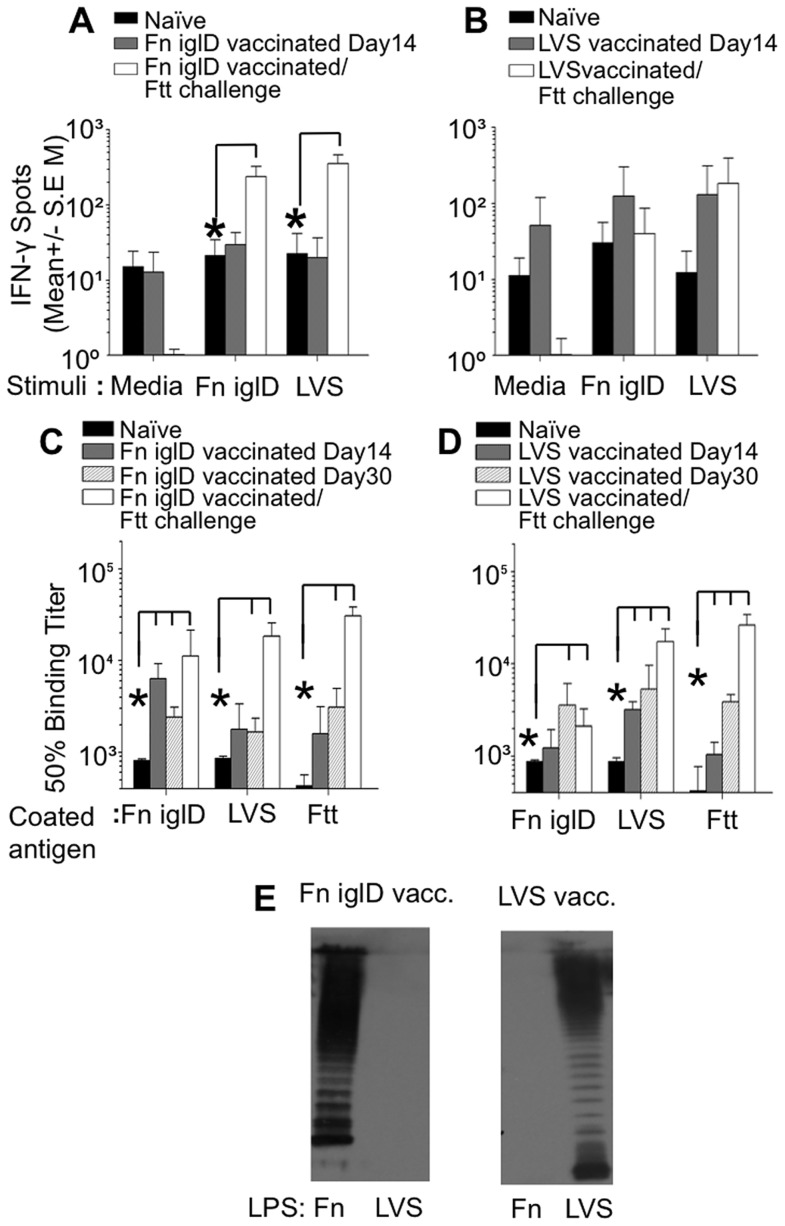
Cellular and humoral responses to *Francisella* spp. in vaccinated NHP. PBMCs were prepared from (**A**) Fn *iglD*- and (**B**) LVS-vaccinated NHPs either prior to vaccination (naïve), on day 14 post-vaccination, or 30 days post Ftt pulmonary challenge. 200,000 cells/well were stimulated *ex vivo* with UV-inactivated Fn-*iglD* (2×10^6^ CFU/ml equivalent) or formalin-fixed LVS (1×10^5^ CFU/ml equivalent) or left unstimulated (medium). IFNγ production was measured by ELISPOT. Assays were performed in triplicate. * indicate significantly (p<0.05; Student t test) more cells produced IFN-γ at the time point indicated as compared to levels measured from naive NHP (Day 0) using the same stimuli. Sera from Fn *iglD*- (**C**) and LVS-vaccinated (**D**) NHPs were analyzed pre-vaccination (naïve), on days 14 and 30 post-vaccination, and 30 days post-Ftt pulmonary challenge by ELISA for total Ab against whole cell killed Fn *iglD*, LVS, and Ftt antigen. Responses of individual Fn *iglD*-vaccinated NHPs are shown in Fig. S7 * indicate significantly (p<0.05; Student t test) higher Ab at the time point indicated as compared to levels measured from naive NHP (Day 0) using the same stimuli. (**E**) Sera from a Fn *iglD*- (left) and LVS-vaccinated NHP (right) were analyzed on day 30 post-vaccination for reactivity to Fn LPS and LVS LPS by Western immunoblot. Sera were from AO8245 (Fn *iglD*-vaccinated) and AO8090 (LVS-vaccinated), and equivalent amounts of LPS were loaded in each well.

T cell responses from vaccinated NHPs were evaluated by measuring IFNγ responses of peripheral blood mononuclear cells (PBMC) upon stimulation with either Fn *iglD* or LVS via ELISPOT (Fig. 4A and 4B). PBMCs were collected from all Fn *iglD*- and LVS-vaccinated NHPs prior to vaccination (naïve) and at day 14 post vaccination. Group responses are shown in Fig. 4A & 4B; responses of individual NHPs are shown in Fig. S6. Neither the Fn *iglD*- nor the LVS-vaccinated NHP groups had significant increases (p>0.05 t test) in cellular responses to either LVS or Fn *iglD* at day 14 post vaccination.

PBMCs were also collected from Fn *iglD*- and LVS-vaccinated NHPs that survived pulmonary challenge with Ftt, 30 days post-challenge. PBMCs were collected from all LVS-vaccinated NHPs, but only successfully collected from three of the five surviving Fn *iglD*-vaccinated NHPs (A08036, A08245, and A09393). Group responses are shown in Fig. 4A; individual responses are shown in Fig. S6. The Fn *iglD*-vaccinated NHP group that survived Ftt challenge showed a significant increase in cellular responses to both LVS and Fn *iglD* stimulation. In contrast, the LVS-vaccinated NHP group that survived Ftt challenge showed a similar cellular response upon LVS stimulation to that seen prior to Ftt challenge, and no response to Fn *iglD* stimulation. Individually, all three Fn *iglD* vaccinated NHPs tested (AO8036, AO8245, AO9393) exhibited enhanced cellular responses post-Ftt challenge to both Fn *iglD* and LVS (Fig. S6), whereas only one of four LVS-vaccinated NHPs (AO7746) mounted an enhanced cellular response post-Ftt challenge to LVS and Fn *iglD*. These results suggest that Fn *iglD* vaccination of NHPs primes T cells that provide a robust response upon challenge with Ftt.

### Identification of immunodominant humoral antigens in Fn *iglD*-vaccinated rats and NHPs

The humoral response in vaccinated NHPs was evaluated by ELISA against whole killed bacteria. Total IgG responses to whole cell Fn *iglD*, LVS, and Ftt were determined for both Fn *iglD*- and LVS-vaccinated NHPs (Fig. 4B). While the strongest initial response (Day 14) was toward Fn in Fn *iglD*-vaccinated animals and toward LVS in LVS-vaccinated animals, cross-reactive antibodies to LVS or Fn and Ftt were induced in vaccinated NHPs at day 30. Increases in serum antibody titers were seen against all three subspecies in Fn *iglD*-vaccinated NHP after challenge with Ftt. Interestingly, a comparison of the individual serum antibody titers in the Fn *iglD* vaccinated NHPs (Fig. S7) revealed lower levels of Fn-specific serum IgG 30 days post vaccination in the animal that succumbed to disease (A08070) than in the 5 other animals that survived challenge. This suggests that anti-Fn antibodies may represent a correlate of protection in this model with this vaccine, although further studies with increased sample size are needed to determine this.

To determine if a humoral response to LPS OAg was induced in vaccinated NHPs, we performed Western immunoblot analyses of sera from Fn *iglD*- and LVS-vaccinated NHPs against purified LPS from Fn and LVS (Fig. 4E). The serum from a Fn *iglD*-vaccinated NHP (AO8245; Fig. 4E) reacted strongly with Fn LPS and not at all with LVS LPS, whereas a LVS-vaccinated NHP (AO8090) reacted with LVS LPS and not at all with Fn LPS. These results again confirm that the OAg of Fn and LVS/Ftt are distinct and demonstrate that in NHP, humoral responses directed towards Fn OAg do not crossreact with Ftt (LVS) OAg, and *vice versa*. Moreover, the NHP humoral response in both vaccinated groups appears primarily directed to OAg associated with LPS, and not the OAg capsule, as was seen in vaccinated rats (Fig. S2).

To identify immunodominant humoral protein antigens associated with Fn *iglD* vaccination in rats and NHP, sera from vaccinated animals was subjected to a *Francisella* proteome microarray [27]. The 10 most reactive antigens with NHP sera at day 28 post pulmonary vaccination with Fn *iglD* (compared to naïve NHP sera) are listed in Table 2; antigens are listed by the corresponding homologous ORF in Ftt. Comparisons are made to the immunodominant antigens with NHP sera at day 28 post subcutaneous vaccination with LVS, as well as to the immunodominant antigens with rat sera at day 28 post pulmonary vaccination with Fn *iglD* (compared to naïve rat sera) (from Fig. 2). The immunodominant antigens identified with Fn *iglD*-vaccinated NHP sera that are also one of the 20 most reactive antigens with the other sera are noted with a “+” (Table 2). A comparison is also made to immunodominant antigens identified with sera from mice vaccinated with killed LVS delivered with adjuvant intramuscularly from a previous study [27]; this vaccination regimen partially protected mice (40% protection) against challenge with 6 CFU Ftt delivered subcutaneously.

**Table 2:** Immunodominant antigens reactive with Fn *iglD* vaccinated NHP sera.

	vaccine	Fn *iglD* (D28)	LVS (live) (D28)	Fn *iglD* (D28)	LVS (killed) (D55)^1^	Circulating antigen?^2^
	route	pulmonary	intradermal	pulmonary	intramuscular	
	animal	NHP	NHP	rat	mouse	mouse
FTT	gene name					
FTT0901	*lpnA*	+	+	+	+	+
FTT1484	*aceF*	+	+	+	+	+
FTT1103	-	+	+	+	+	
FTT1539	-	+	+	+	+	
FTT0472	*accB*	+	+	+	+	
FTT1696	*groL*	+	+		+	+
FTT1747	-	+	+		+	+
FTT0863	-	+	+		+	
FTT0975	-	+	+		+	
FTT1636	*lolA*	+				

1 from Ref [27], BALB/c mice were vaccinated intramuscularly with killed LVS mixed with immune stimulating complexes and CpG on days 0, 28, and 49.

2 identified in Ref [28] as an antigen that is secreted or shed into serum during Ftt infection of BALB/c mice.

It is notable that the top five immunodominant humoral protein antigens recognized by NHP vaccinated with Fn *iglD* via the pulmonary route are also immunodominant antigens following vaccination with Fn *iglD* in rats via the pulmonary route, or following vaccination with LVS (live) in NHP via the intradermal route or LVS (killed) in mice via the intramuscular route. In fact, nine of the top ten immunodominant protein antigens are shared between NHP vaccinated with either Fn *iglD* or LVS, and mice vaccinated with LVS (Table 2). Four of these antigens were identified as being secreted or shed during Ftt infection of mice [28]. The immunodominant antigens reactive with either NHPs or rats vaccinated via the pulmonary route with Fn *iglD* shared the top five antigens in common.

All of the vaccinated NHPs and rats in these groups survived pulmonary challenge with >1000 CFU Ftt, with the exception of one NHP (A08070). While anti-whole cell Fn humoral reactivity was lower in A08070 (Fig. S7), humoral reactivity to these ten specific immunodominant antigens was not obviously deficient. Further studies with larger sample sizes are needed to determine if specific humoral response(s) represent correlate(s) of protection against pulmonary Ftt challenge. These results demonstrate that a core set of immunodominant antigens stimulate the humoral response during vaccination, regardless of route, animal host, or *Francisella* subspecies.

## Discussion

Ftt acquired through the pulmonary route leads to serious disease with a high mortality rate in humans. Although pneumonic tularemia caused by natural Ftt infection is relatively rare, this bacterium was investigated as a bioweapon by several government programs, and the potential exists for its illicit use against human populations. Because of this, Ftt has been classified as a select biothreat agent, and efforts are underway to develop an effective vaccine against pulmonary exposure to Ftt. *Francisella* subspecies are facultative intracellular bacteria that primarily reside within cells in infected animals, and thus T cell-mediated immunity is an important component of protection against tularemia. However, humoral immunity has also been shown to contribute to protection against *Francisella* infection [29], [30].

In limited studies, LVS vaccination via scarification of humans provided protection against pulmonary Ftt challenge, but the vaccine strain needed to be live rather than killed [7]. Due to questions regarding phase variation, genetic cause of attenuation, and levels of protection afforded, it is questionable whether LVS will be approved for human usage. LVS still serves as a useful model for the stimulation of protective immunity in various animal models of tularemia, and we have shown here that it stimulates protective immunity against pulmonary exposure to Ftt in cynomolgus macaques. The ability of a live attenuated *Francisella* strain such as LVS to protect against pulmonary Ftt exposure indicates that a genetically defined live attenuated *Francisella* strain may constitute the optimal tularemia vaccine, especially since no protective subunit antigens have yet been identified.

*F. novicida* (Fn) is closely related to *F. tularensis* [31]; although it is officially classified as a separate species, it is frequently referred to as a subspecies of *F. tularensis* because of this close genetic relationship. Fn has generally been discounted as a potential vaccine against Ftt because although vaccination of mice with live attenuated Fn strains can induce good protection against homologous pulmonary challenge with wildtype Fn, it provides no protection against pulmonary challenge with Ftt. However, vaccination of mice with live attenuated Ftt strains also provides little protection against pulmonary challenge with Ftt, as we have shown here, suggesting that the mouse model may not be appropriate for the assessment of vaccine potential due to its extreme sensitivity to *Francisella* infections. Indeed, mice are highly susceptible to both Fn and LVS infections, despite the low virulence of these strains in humans. We would argue that tularemia vaccine development for humans requires animal models that reflect human sensitivities to the various *Francisella* species/subspecies.

The Fischer 344 rat reflects the relative sensitivities of humans to *Francisella* infections, in that it is sensitive to Ftt pulmonary infections, but resistant to pulmonary Fn infections (approximately 10^4^-fold difference in LD_50_; [11]). Importantly, rats that survive Fn infection are protected against subsequent pulmonary challenge with Ftt, demonstrating the efficacy of Fn as a tularemia vaccine platform in this model [11]. In the current study, utilizing the same attenuating mutation (*iglD*) in either the Fn or Ftt background, we showed that vaccination of rats with either Ftt *iglD* or Fn *iglD* strain provided protection against Ftt pulmonary challenge. This demonstrates that, at least in this model, there is no disadvantage to utilizing Fn instead of Ftt as the vaccine platform. With a single oral vaccination of Fn *iglD* high levels of protection (83%) were achieved against pulmonary Ftt challenge. Even higher levels of protection (100%) were achieved against Ftt pulmonary challenge by a single pulmonary (intratracheal) vaccination with Fn *iglD*. We have previously shown that a Fn strain containing a different attenuating mutation (*iglB*) that prevents intramacrophage replication can also protect Fischer 344 rats against pulmonary Ftt challenge when administered by either pulmonary or oral vaccination [23]. The Fn *iglD* strain used in the current study appears to induce higher levels of protection in rats, although further direct comparative studies would be needed to establish the relative protective capacities of these two potential vaccine candidates. Regardless, the successes of attenuated Fn strains to protect rats against Ftt pulmonary challenge indicate the promise of this platform in tularemia vaccine development.

Given their close genetic relatedness with humans, non-human primates are considered to be valuable models of disease, especially for vaccine development. The cynomolgus macaque is susceptible to pulmonary Ftt infection, which results in a fatal systemic disease similar to that seen in humans ([15]; manuscript in preparation). Additionally, we show here that LVS vaccination via the subcutaneous route protects these NHPs against pulmonary Ftt challenge, similar to humans. Importantly, pulmonary vaccination of cynomolgus macaques with a single dose of Fn *iglD* also provided high levels of protection (83%) against aerosol challenge with Ftt (>1000 CFU). This is the first demonstration of efficacy of a defined live attenuated vaccine strain against aerosol Ftt exposure in a NHP. In this model, indicators of disease progression include increased respiration rate, elevated serum CRP levels, and high bacterial organ burdens. Vaccination of the NHPs with Fn *iglD* resulted in reduction in all these indicators following pulmonary Ftt challenge, similar to vaccination with LVS.

Analyses of the sera from vaccinated animals indicated that the immunodominant protein antigens recognized by NHPs vaccinated with Fn *iglD* were largely the same (9 of 10) as those in NHPs vaccinated with LVS, suggesting that humoral immunodominant protein antigens are conserved between Fn and Fth/Ftt. Four of these antigens (FTT0472, FTT0975, FTT1484, FTT1696) were also identified as within the top 25 immunoreactive antigens using the same proteome microarray with convalescent sera from human patients with Ft infections [32]. Four additional of these antigens (FTT0901, FTT1103, FTT1539, FTT0863) were identified by 2-D immunoblotting as immunoreactive with convalescent sera from human patients with Fth infections [33] These immunodominant antigens may provide a guide to tularemia subunit vaccine development in the future. Notably, Fn *iglD* vaccination of NHPs induced strong reactivity to Fn LPS but no cross-reactivity to LVS/Ftt LPS, suggesting that protection against Ftt infection by this vaccine does not include antibodies against the LPS OAg.

For the near term, the Fn *iglD* strain has several characteristics that make it an attractive tularemia vaccine candidate. First, Fn exhibits low virulence in healthy humans, making it an inherently safer vaccine platform than the high virulence Ftt and Fth strains. Second, because of the inherent low virulence, Fn is exempt from select agent status, unlike Ftt and Fth, which allows for ease of use, transport, genetic manipulation, etc, without need for high level biocontainment facilities. Third, the defined *iglD* mutation prevents intracellular replication in permissive host cells and virulence in permissive animal models, resulting in a highly attenuated and inherently safer strain. Finally, Fn is more amenable to genetic manipulations than Ftt or Fth [34], which facilitates the further development of this vaccine platform to enhance efficacy and provide protection against heterologous antigens.

## Materials and Methods

### Ethics statement

This study was performed in strict accordance with the recommendations in the Guide for the Care and Use of Laboratory Animals of the National Institutes of Health. Animal protocols involving rodents were approved by the University of Texas at San Antonio Institutional Animal Care and Use Committee (IACUC) under protocol MU009(RA). The animal protocol for NHPs was approved by the Lovelace Respiratory Research Institute IACUC under protocol FY09-126. LRRI has attending veterinarians and animal care staff that are available 24 hrs a day, 7 days a week to assist in any animal care issues. All study animals were housed individually in primate cages, and food and water were supplied *ad libitum* except when animals were removed from their cages for study procedures. Harlan Teklad Certified 20% Monkey Diet (W) 2050C was fed to the animals daily, and for daily food enrichment, each animal received ¼ cup of fruit or vegetable prepared by enrichment technicians. The Study Director and the Attending Veterinarian discussed the study protocol and agreed upon scientifically appropriate analgesic, anesthetics and tranquilizing drugs prior to submission of the protocol to the LRRI IACUC. All necessary efforts were made to minimize discomfort, distress, pain, or injury to study animals. To ameliorate suffering, NHPs were conditioned to a restraint collar and restraint chair for sampling, and an implanted transponder allowed for non-invasive measurement of body temperature and respiration rate by a hand-held device. NHPs were anesthetized under the guidance of a veterinarian or a registered veterinary technician (RVT) to perform the following procedures: general physical examination, collar placement, aerosol challenge and euthanasia. NHPs were kept warm while under anesthesia with delta phase heating pads. All anesthetic doses were determined from the most recent weight, and NHPs were constantly monitored for respiration and recovery by a veterinarian or RVT. Aerosol challenge doses of *F. tularensis* were delivered to anesthetized NHPs in a head-only exposure chamber, during which they were breathing freely. The method of euthanasia selected for these studies was administration of barbiturate overdose via intravenous or intramuscular injection following notification of and authorization from the Study Director or the Attending Veterinarian. Euthanasia is always administered to individual animals in a separate area out of the sight of other surviving study animals.

### Strains and media

The Fn *iglD* strain KKF37 [19] is isogenic with wildtype Fn strain U112 and the Ftt *iglD* strain KKT8 is isogenic with wildtype Ftt strain Schu S4. The Ftt *iglD* strain had both copies of *iglD* (*iglD1 iglD2*) inactivated by a Group II intron targeted to *iglD*, as described in [35]. *Francisella* strains were grown in tryptic soy broth (TSB) (BD Biosciences) supplemented with 0.1% (w/v) L-cysteine (Fisher Scientific) and sodium metabisulfite (Sigma), iron sulfate (Mallinckrodt), and sodium pyruvate, all at 250 µg/ml final concentration, or Chamberlain's defined medium [36].

### Mouse and rat virulence assays

BALB/c mice were inoculated intranasally as described previously [37]. Female Fischer 344 rats were inoculated intratracheally or orally as described previously [23]. After sacrifice, spleens were collected for T cell recall assays, which were performed as previously described [23]. All animal protocols have been approved by the University of Texas at San Antonio Institutional Animal Care and Use Committee and Institutional Biosafety Committee.

### Non-human primate virulence assays

Male and female cynomolgus macaques (*Macaca fascicularis*, Vietnamese origin, approximately 2 yrs old) were received from Covance (Alice, TX). LVS vaccinees were immunized with ∼1.8×10^8^ LVS organisms by the subcutaneous route. Fn-*iglD* vaccinees were immunized via bronchoscope with a dose of 1×10^8^ CFU. Control animals were untreated. This animal protocol was approved by the Lovelace Respiratory Research Institute IACUC. For challenges, *Ftt* Schu S4 was nebulized using a Collison MRE-3 nebulizer and delivered to the anesthetized NHPs in a head-only exposure chamber. The aerosol was sampled directly and viable *Ftt* CFU were confirmed by quantitative bacterial culture. Blood was collected via venipuncture and analyzed by culture on chocolate agar, and serum was analyzed for CRP, LDH, BUN, ALT and AST. At necropsy, tissues were taken and analyzed for the presence of *Ftt* by quantitative culture.

### Serum analyses

Peripheral Blood Mononuclear Cells (PBMC) were prepared from NHP blood by gradient separation (Lymphoprep, Accurate Chemical and Scientific Corp.). For measurement of cellular responses, PBMCs (200,000/well) were added to ELISPOT plate wells (Mabtech #3420M-2HW-Plus) that had been coated with anti-monkey IFNγ (15 µg/mL, clone GZ-4, 100 µL/well). UV-inactivated Fn-*iglD* (2×10^6^ CFU/ml) or formalin-fixed LVS (1×10^5^ CFU/ml) were then added as stimuli. Plates were incubated for 20 hours (37°C; 5% CO_2_), washed with PBS, followed by the addition of biotinylated mouse IgG1 anti-monkey IFNγ antibody (clone 7-B6-1; 0.1 µg/well) and incubation at RT for 2 h. Plates were washed with PBS, 100 µl/well streptavidin-horseradish peroxidase (HRP) was added (1∶1000 dilution in PBS 0.5% FCS), and plates were then incubated at RT for 1 hour. Plates were washed and HRP substrate (100 µl/well) was added. After 20–40 min of incubation at RT, plates were rinsed with water, dried, and read on a CTL Immunospot reader.

Serum ELISAs were run to obtain 50% binding titers to UV-inactivated Fn *iglD*, LVS, or *Ftt* (10^6^ cells/well). Secondary antibody specific for either rat total antibody, IgG1, or IgG2a (Southern Biotech, Birmingham, AL), or monkey IgG (KPL, Gaithersburg MD) was added for 1 hr. After wash, TMB substrate (BD Biosciences) was added. Sera were also evaluated by *Francisella* proteome microarray (Antigen Discovery Inc., [27]). Sera were analyzed for reactivity against purified Fn or LVS LPS (kind gift of J. Gunn) by Western immunoblot, utilizing either anti-rat (GE Healthcare) or anti-monkey (KPL) HRP conjugate. Each well contained either 50 µg (NHP) or 75 µg (rat) purified LPS.

## Supporting Information

Figure S1 **A.** **Fn** ***iglD*** **and Ftt** ***iglD*** **strains are defective for intramacrophage replication.** *F. novicida* strains U112 (wildtype) and KKF37 (Fn *iglD*), and *F. tularensis* subsp. *tularensis* strains Schu S4 (wildtype) and KKT8 (Ftt *iglD*; because Ftt has two copies of *iglD*, this strain is actually *iglD1 iglD2*) were inoculated at an MOI of ∼10∶1 into J774 cells, and intracellular bacteria were enumerated at 3 and 24 h. The assay was performed in triplicate. *P-value = 0.0083, **P-value = 0.0011. **B.** **Fn** ***iglD*** **and Ftt** ***iglD*** **strains are attenuated for virulence in mice.** *F. novicida* strains U112 (wildtype) or KKF37 (Fn *iglD*), and *F. tularensis* subsp. *tularensis* strains Schu S4 (wildtype) and KKT8 (Ftt *iglD* were inoculated intranasally into groups of 5 female BALB/C mice and approximate LD_50_ calculated based on survival at 30 days. All mice survived inoculation with the highest doses of Fn *iglD* (9.7×10^8^ CFU) and Ftt *iglD* (4.8×10^6^ CFU).
(TIFF)

Figure S2 **Reactivity of Fn** ***iglD*** **- and LVS-vaccinated rats to Fn and LVS LPS.** Sera from rats vaccinated intratracheally with either 10^7^ CFU Fn *iglD* (**A**) or 10^7^ CFU LVS (**B**) were collected 28 days post vaccination, and evaluated for reactivity against purified LPS from Fn strain U112 (Fn) or Fth strain LVS by Western immunoblot.
(TIFF)

Figure S3 **Intratracheal vaccination with Fn** ***iglD*** **induces cellular immunity in rats.** Fischer 344 rats (n = 3 per group) were vaccinated i.t. with 10^7^ CFU Fn *iglD* or mock-vaccinated with PBS and rested for 28 days. Rats were sacrificed and spleens collected to prepare single-cell suspensions. Splenocytes (10^6^ cells/well) were cultured in triplicate for 72 hrs with either 1 µg of unrelated antigen hen egg lysozyme (HEL), or two different doses (10^5^ or 10^6^ CFU) of UV-inactivated Fn *iglD*. Supernatants were collected and assayed by ELISA for IFN-γ production. Assays were performed in triplicate. *p<0.05 Student t test.
(PDF)

Figure S4 **Serum markers in vaccinated NHP challenged with pulmonary** ***Ftt*** **.** Groups of cynomolgus macaques were vaccinated with 10^8^ CFU Fn *iglD* delivered via bronchoscopy (n = 6; open circles), or 10^8^ CFU LVS delivered subcutaneously (n = 4; filled squares), or mock vaccinated (n = 4; filled triangles). 35 days post vaccination NHP were challenged with ∼1000 CFU Ftt via head-only aerosol inhalation; actual presented doses were determined (Table S1). NHP were monitored at intervals shown for 30 days post-challenge for (**A**) blood urea nitrogen (BUN), (**B**) lactate dehydrogenase (LDH), (**C**) serum alanine transaminase (ALT), and (**D**) aspartate aminotransferase (AST) levels. The single Fn *iglD* vaccinated NHP that succumbed to pulmonary Ftt challenge was not included in A, B, C, and D.
(TIFF)

Figure S5 **Organ burdens of individual vaccinated NHPs at autopsy.** Bacterial burdens (Ftt) were determined in spleen, lung, mesenteric lymph nodes (MesLN), liver, and trachea-bronchial lymph nodes (TBLN) at the time of euthanasia for **A.** Fn *iglD*-vaccinated and **B.** LVS- vaccinated NHPs. Euthanasia was at day 30 post-Ftt challenge, with the exception of animal A08070, which was euthanized on day 9 post Ftt challenge. The limit of detection (“l.o.d.”) was 70 CFU/g, shown by line.
(TIFF)

Figure S6 **Cellular responses of individual vaccinated NHPs.** PBMCs were prepared from (**A**) Fn *iglD*- and (**B**) LVS-vaccinated NHPs either prior to vaccination (naïve), on day 14 post-vaccination (“Fn iglD/LVS vaccinated day 14”), or 30 days post Ftt pulmonary challenge (“Fn iglD/LVS vaccinated Ftt challenge”). 200,000 cells/well were stimulated *ex vivo* with UV-inactivated Fn-*iglD* (2×10^6^ CFU/ml equivalent) or formalin-fixed LVS (1×10^5^ CFU/ml equivalent) or left unstimulated (medium). IFNγ production was measured by ELISPOT. Assays were performed in triplicate. Results are indicated by NHP subject number followed by stimulant. In **A**, insufficient PBMC collection prevented assay of A08077 and A08532 after challenge with Ftt, and A08070 did not survive Ftt challenge, thus the “Fn iglD vaccinated/Ftt challenge” values are missing from these NHPs. Dotted line indicates limit of detection (1 spot) in individual well.
(TIFF)

Figure S7 **Humoral responses to** ***Francisella*** **spp. in individual vaccinated NHPs.** Sera from individual Fn *iglD*-vaccinated NHPs were analyzed pre-vaccination (naïve), on days 14 and 30 post-vaccination, and 30 days post-Ftt pulmonary challenge by ELISA for total Ab against whole cell killed Fn *iglD*, LVS, and Ftt antigen.
(TIFF)

Table S1 **Ftt aerosol challenge doses in NHPs.**
(DOCX)
