# Supplementary figures and images for: Live Attenuated Francisella novicida Vaccine Protects against Francisella tularensis Pulmonary Challenge in Rats and Non-human Primates
Source: PLoS Pathog. 2014 Oct 23;10(10):e1004439. doi: 10.1371/journal.ppat.1004439 (PMC4207810; doi:10.1371/journal.ppat.1004439)

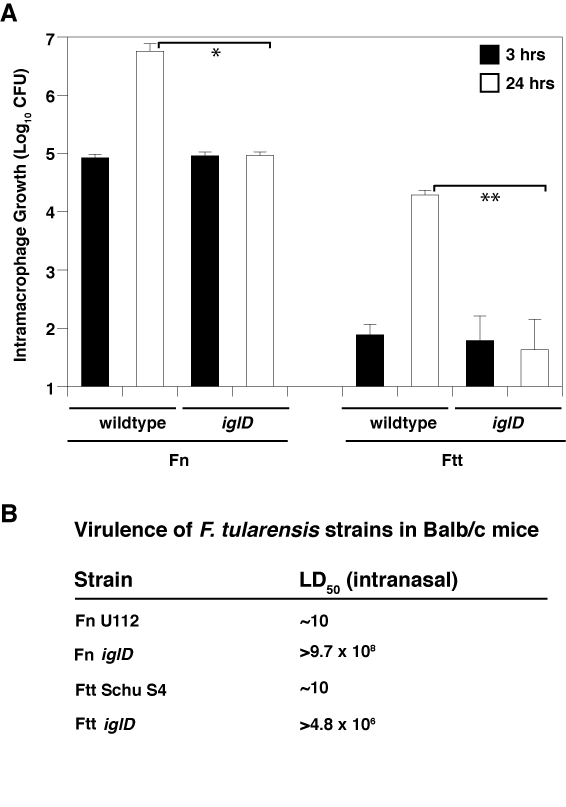

Supplement: Figure S1 — A. Fn iglD and Ftt iglD strains are defective for intramacrophage replication. F. novicida strains U112 (wildtype) and KKF37 (Fn iglD), and F. tularensis subsp. tularensis strains Schu S4 (wildtype) and KKT8 (Ftt iglD; because Ftt has two copies of iglD, this strain is actually iglD1 iglD2) were inoculated at an MOI of ∼10∶1 into J774 cells, and intracellular bacteria were enumerated at 3 and 24 h. The assay was performed in triplicate. *P-value = 0.0083, **P-value = 0.0011. B. Fn iglD and Ftt iglD strains are attenuated for virulence in mice. F. novicida strains U112 (wildtype) or KKF37 (Fn iglD), and F. tularensis subsp. tularensis strains Schu S4 (wildtype) and KKT8 (Ftt iglD were inoculated intranasally into groups of 5 female BALB/C mice and approximate LD50 calculated based on survival at 30 days. All mice survived inoculation with the highest doses of Fn iglD (9.7×10^8^ CFU) and Ftt iglD (4.8×10^6^ CFU). (TIFF) [file ppat.1004439.s001.tiff]

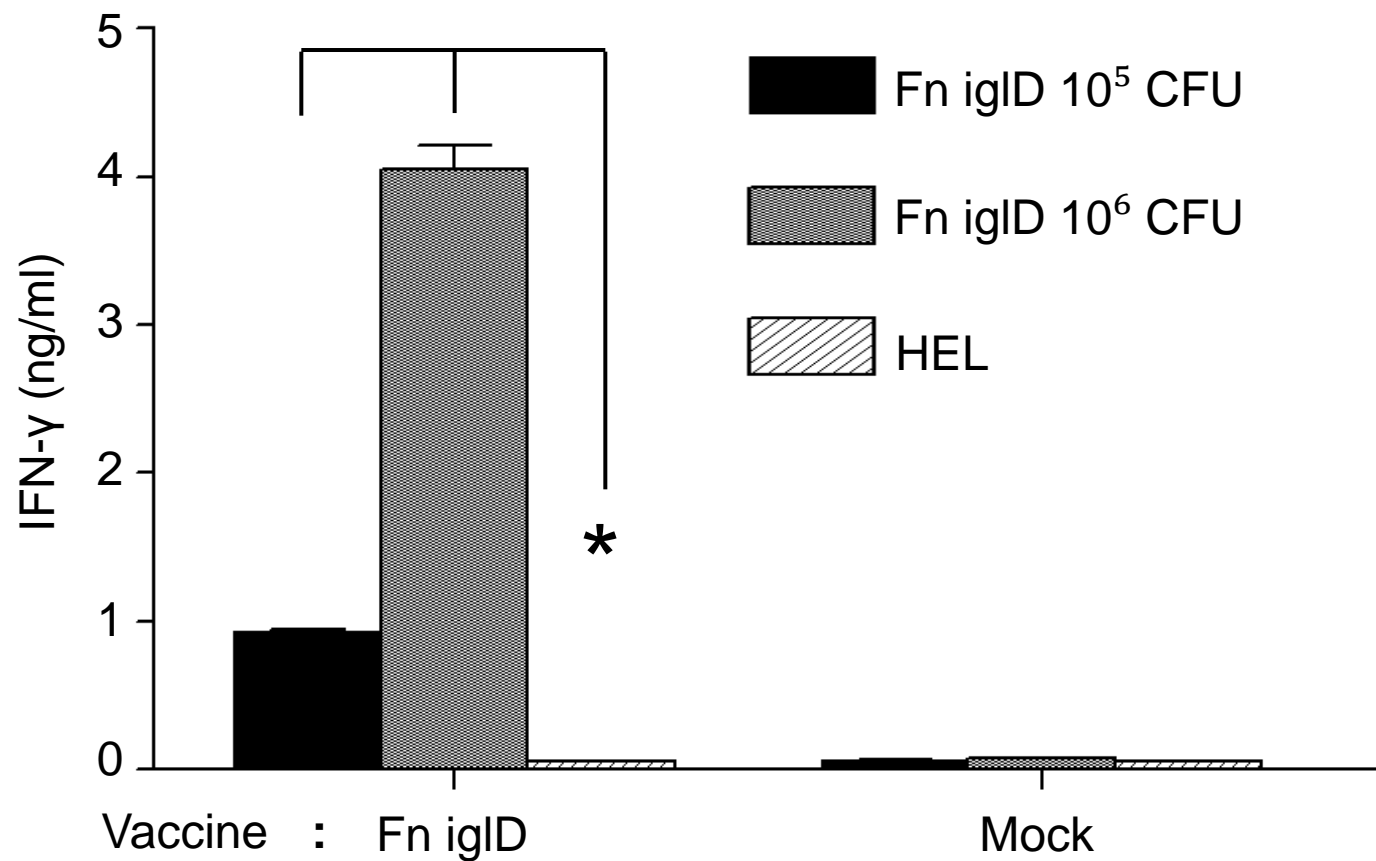

Supplement: Figure S3 — Intratracheal vaccination with Fn iglD induces cellular immunity in rats. Fischer 344 rats (n = 3 per group) were vaccinated i.t. with 107 CFU Fn iglD or mock-vaccinated with PBS and rested for 28 days. Rats were sacrificed and spleens collected to prepare single-cell suspensions. Splenocytes (106 cells/well) were cultured in triplicate for 72 hrs with either 1 µg of unrelated antigen hen egg lysozyme (HEL), or two different doses (105 or 106 CFU) of UV-inactivated Fn iglD. Supernatants were collected and assayed by ELISA for IFN-γ production. Assays were performed in triplicate. *p<0.05 Student t test. (PDF) [file ppat.1004439.s003.pdf]
