# Supplementary material for: Live Attenuated Francisella novicida Vaccine Protects against Francisella tularensis Pulmonary Challenge in Rats and Non-human Primates
Source: PLoS Pathog. 2014 Oct 23;10(10):e1004439. doi: 10.1371/journal.ppat.1004439 (PMC4207810; doi:10.1371/journal.ppat.1004439)
Supplement: Table S1 — Ftt aerosol challenge doses in NHPs. (DOCX) [file ppat.1004439.s008.docx]

**Table S1: Ftt Aerosol Challenge Doses***

| **Animal Number** | **Gender** | **Vaccinated with** | **Ftt aerosol challenge dose (CFU)** |
| --- | --- | --- | --- |
| A08223 | M | mock | 844 |
| A08374 | M | mock | 832 |
| A08060 | F | mock | 544 |
| A07796 | F | mock | 770 |
| A08371 | M | LVS | 452 |
| A09084 | M | LVS | 2440 |
| A08090 | F | LVS | 1610 |
| A07746 | F | LVS | 2230 |
| A08532 | M | Fn *iglD* | 2500 |
| A08036 | M | Fn *iglD* | 5060 |
| A08245 | M | Fn *iglD* | 4810 |
| A08077 | F | Fn *iglD* | 2580 |
| A08070 | F | Fn *iglD* | 3690 |
| A09393 | F | Fn *iglD* | 3410 |

* aerosol presented doses were determined by the following formula: 𝑃𝑟𝑒𝑠𝑒𝑛𝑡𝑒𝑑 𝐷𝑜𝑠𝑒 (𝐶𝐹𝑈)= 𝐶𝐹𝑈/𝐿×𝐼𝑛ℎ𝑎𝑙𝑒𝑑 𝑉𝑜𝑙𝑢𝑚𝑒 (𝐿). The total time that the animal is exposed is used along with the plethysmography data to determine the volume of air inhaled.  The inhaled volume is measured with real-time plethysmography and the CFU/L inhaled is obtained by sampling a portion of the aerosol provided to the animal.  The animal is challenged with approximately 16 L air/minute containing the viable aerosol with 5 L/min sampled to an all glass impinger (AGI).
